# Supplementary figures and images for: Assembly of chloroplast genomes with long- and short-read data: a comparison of approaches using Eucalyptus pauciflora as a test case
Source: BMC Genomics. 2018 Dec 29;19:977. doi: 10.1186/s12864-018-5348-8 (PMC6311037; doi:10.1186/s12864-018-5348-8)

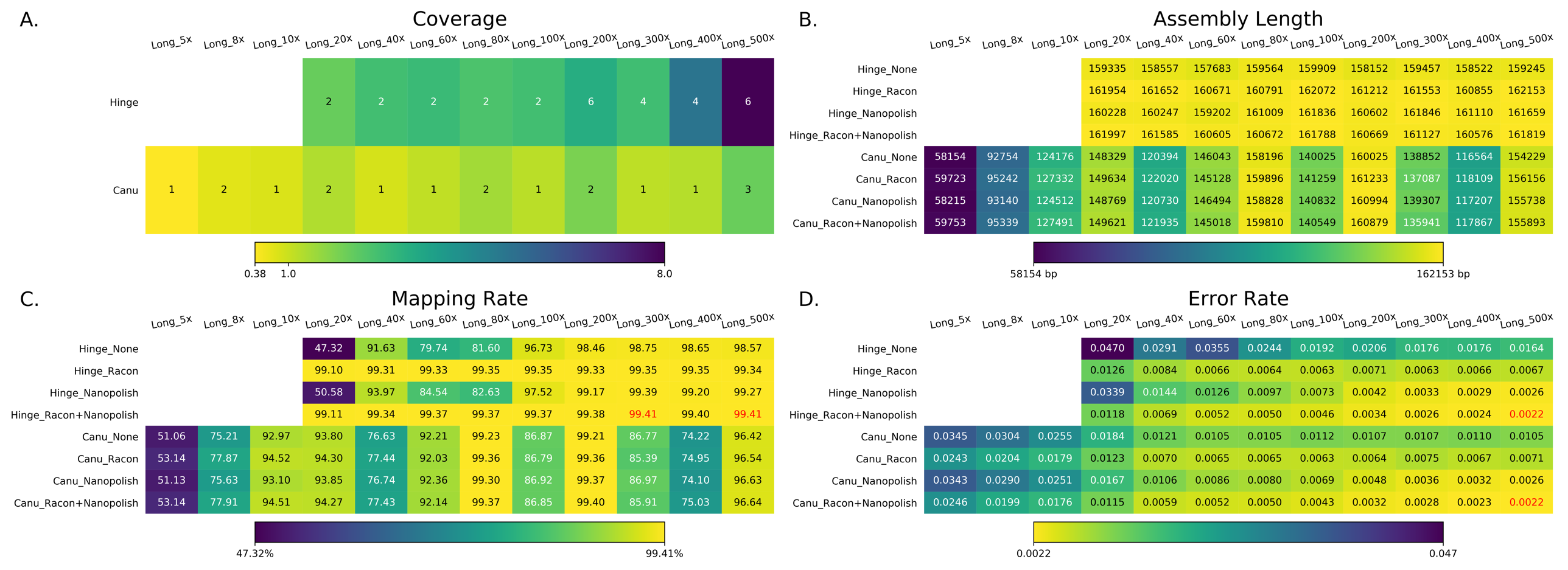

Supplement: Supplementary file 2 — Figure S1. The summary of long-read-only assemblies. Long_5x indicates the 5x coverage of long-read was used in the assembly. Hinge failed to assemble genome with <20x coverage. None, Racon, Nanopolish and Racon+Nanopolish mean the different genome polishing pipeline. A. The total coverage of the chloroplast genome across all contigs output by the assembler. The number is the number of contigs of each assembly, whereas the heatmap is the genome coverage (it could be over 100% if some duplications exist). B. The assembly length of different assemblies after manual curation (e.g. removing duplicate regions). C. The mapping rate of validation reads to the assemblies after manual curation. D. The average per-base error rate of validation reads mapped to each manually-curated genome assembly. (PNG 1920 kb) [file 12864_2018_5348_MOESM2_ESM.png]

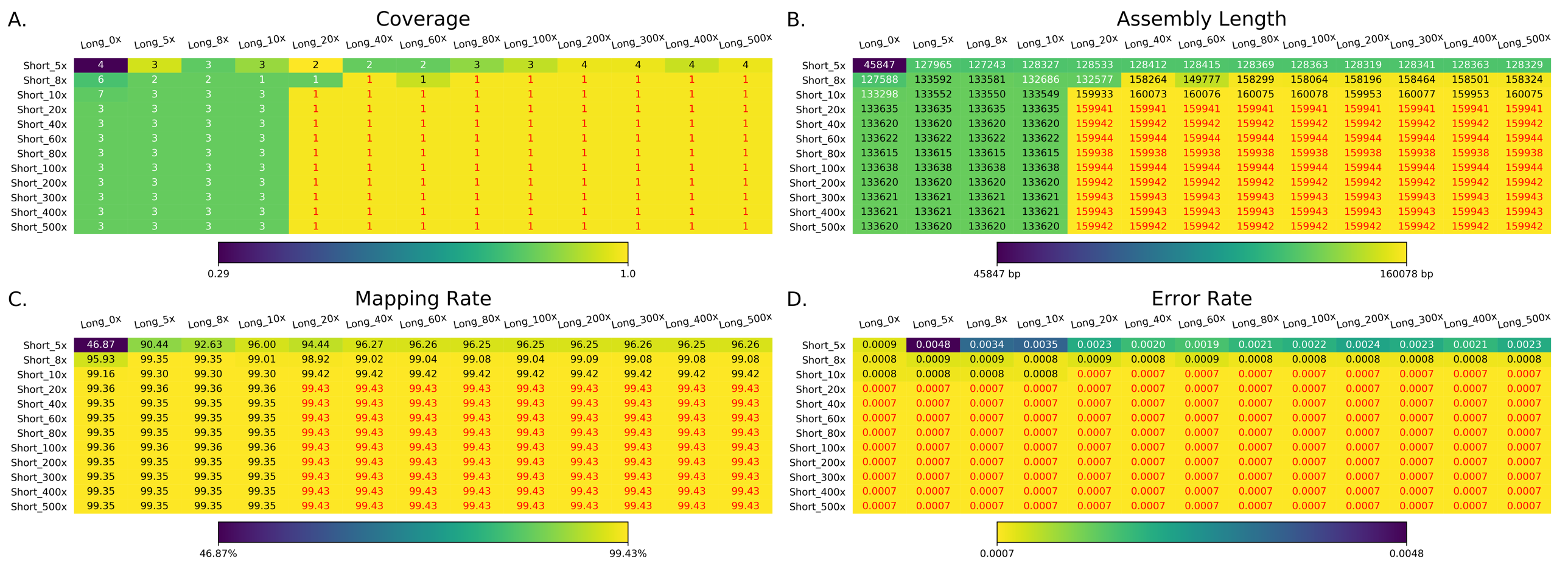

Supplement: Supplementary file 3 — Figure S2. The summary of short-read-only and hybrid assemblies with Karect short-read correction. Long and Short indicate the coverage of this assembly. A. The total coverage of the chloroplast genome across all contigs output by the assembler. The number is the number of contigs of each assembly, whereas the heatmap is the genome coverage (it could be over 100% if some duplications exist). Numbers marked with red contained a single contig covering the whole chloroplast genome. The heatmap color is reversed compared to the Fig. 1 to make the color in all figure panel A show consistence. B. The assembly length of different assemblies after manual curation (e.g. removing duplicate regions). Numbers marked with red denote assemblies with the expected length, in the range 155,938 bp–155,945 bp. C. The mapping rate of validation reads to the assemblies after manual curation. Assemblies with highest mapping rate (99.43%) are marked with red. D. The average per-base error rate of validation reads mapped to each manually-curated genome assembly. Assemblies with the lowest error rate (0.0007) are marked with red. (PNG 1500 kb) [file 12864_2018_5348_MOESM3_ESM.png]

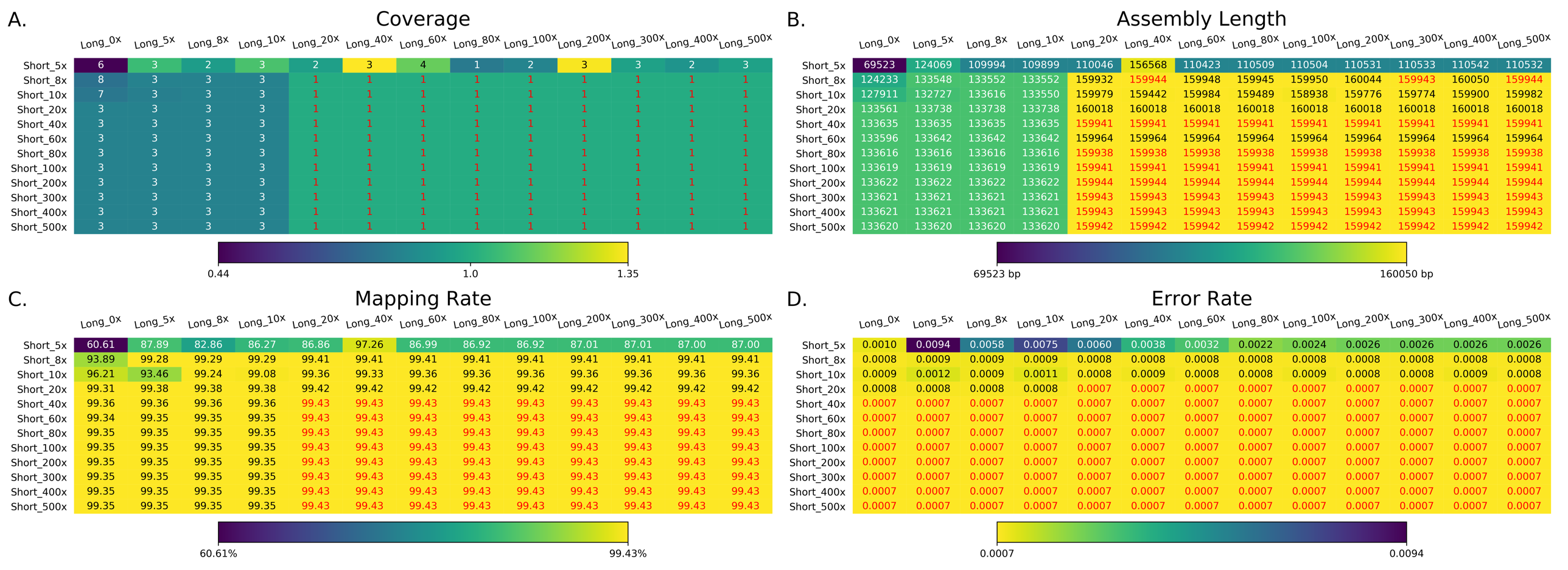

Supplement: Supplementary file 4 — Figure S3. The summary of short-read-only and hybrid assemblies with SPAdes short-read correction. Long and Short indicate the coverage of this assembly. A. The total coverage of the chloroplast genome across all contigs output by the assembler. The number is the number of contigs of each assembly, whereas the heatmap is the genome coverage (it could be over 100% if some duplications exist). Numbers marked with red contained a single contig covering the whole chloroplast genome. The heatmap color is reversed compared to the Fig. 1 to make the color in all figure panel A show consistence. B. The assembly length of different assemblies after manual curation (e.g. removing duplicate regions). Numbers marked with red denote assemblies with the expected length, in the range 155,938 bp–155,945 bp. C. The mapping rate of validation reads to the assemblies after manual curation. Assemblies with highest mapping rate (99.43%) are marked with red. D. The average per-base error rate of validation reads mapped to each manually-curated genome assembly. Assemblies with the lowest error rate (0.0007) are marked with red. (PNG 1550 kb) [file 12864_2018_5348_MOESM4_ESM.png]

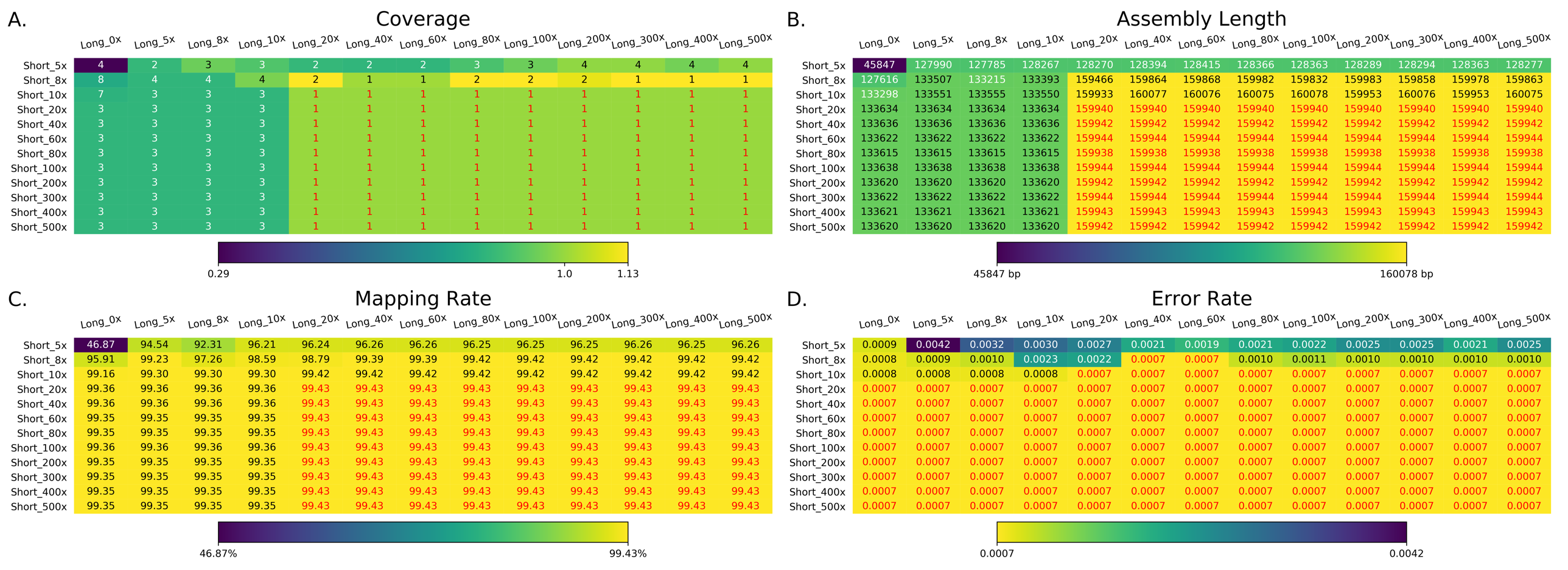

Supplement: Supplementary file 5 — Figure S4. The summary of short-read-only and hybrid assemblies with Karect+SPAdes short-read correction. Long and Short indicate the coverage of this assembly. A. The total coverage of the chloroplast genome across all contigs output by the assembler. The number is the number of contigs of each assembly, whereas the heatmap is the genome coverage (it could be over 100% if some duplications exist). Numbers marked with red contained a single contig covering the whole chloroplast genome. The heatmap color is reversed compared to the Fig. 1 to make the color in all figure panel A show consistence. B. The assembly length of different assemblies after manual curation (e.g. removing duplicate regions). Numbers marked with red denote assemblies with the expected length, in the range 155,938 bp–155,945 bp. C. The mapping rate of validation reads to the assemblies after manual curation. Assemblies with highest mapping rate (99.43%) are marked with red. D. The average per-base error rate of validation reads mapped to each manually-curated genome assembly. Assemblies with the lowest error rate (0.0007) are marked with red. (PNG 1500 kb) [file 12864_2018_5348_MOESM5_ESM.png]

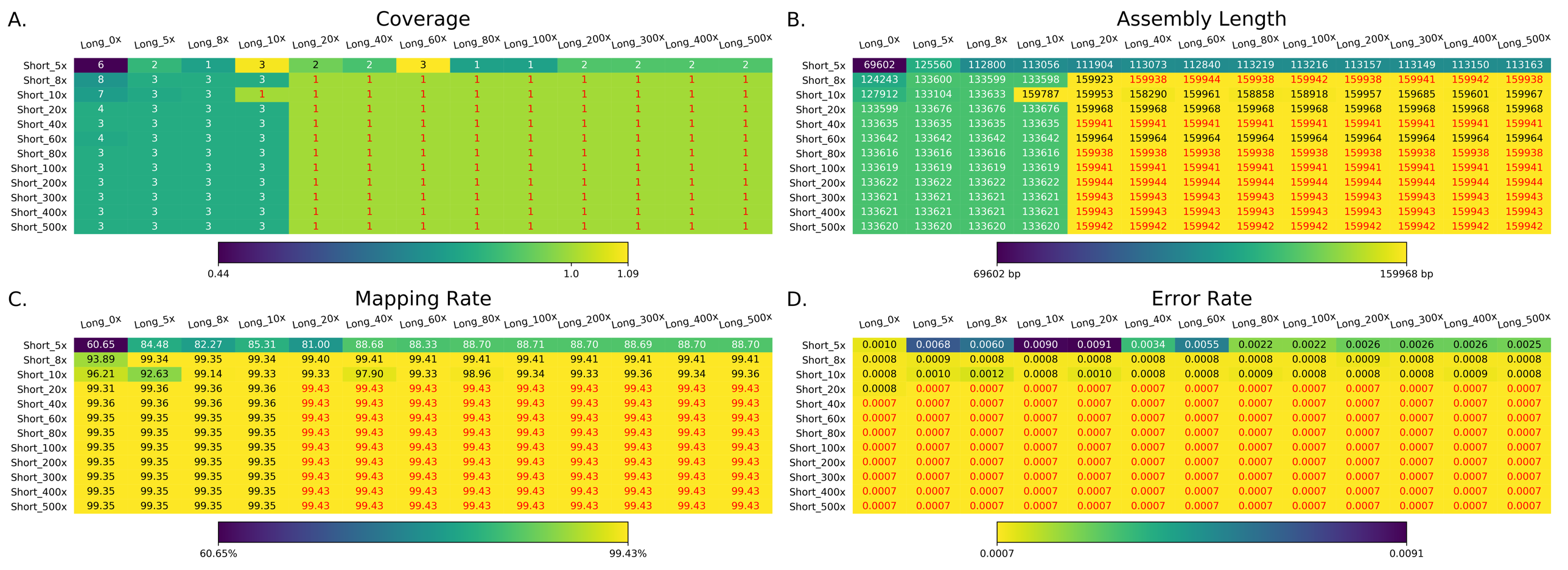

Supplement: Supplementary file 6 — Figure S5. The summary of short-read-only and hybrid assemblies without any short-read correction. Long and Short indicate the coverage of this assembly. A. The total coverage of the chloroplast genome across all contigs output by the assembler. The number is the number of contigs of each assembly, whereas the heatmap is the genome coverage (it could be over 100% if some duplications exist). Numbers marked with red contained a single contig covering the whole chloroplast genome. The heatmap color is reversed compared to the Fig. 1 to make the color in all figure panel A show consistence. B. The assembly length of different assemblies after manual curation (e.g. removing duplicate regions). Numbers marked with red denote assemblies with the expected length, in the range 155,938 bp–155,945 bp. C. The mapping rate of validation reads to the assemblies after manual curation. Assemblies with highest mapping rate (99.43%) are marked with red. D. The average per-base error rate of validation reads mapped to each manually-curated genome assembly. Assemblies with the lowest error rate (0.0007) are marked with red. (PNG 1540 kb) [file 12864_2018_5348_MOESM6_ESM.png]

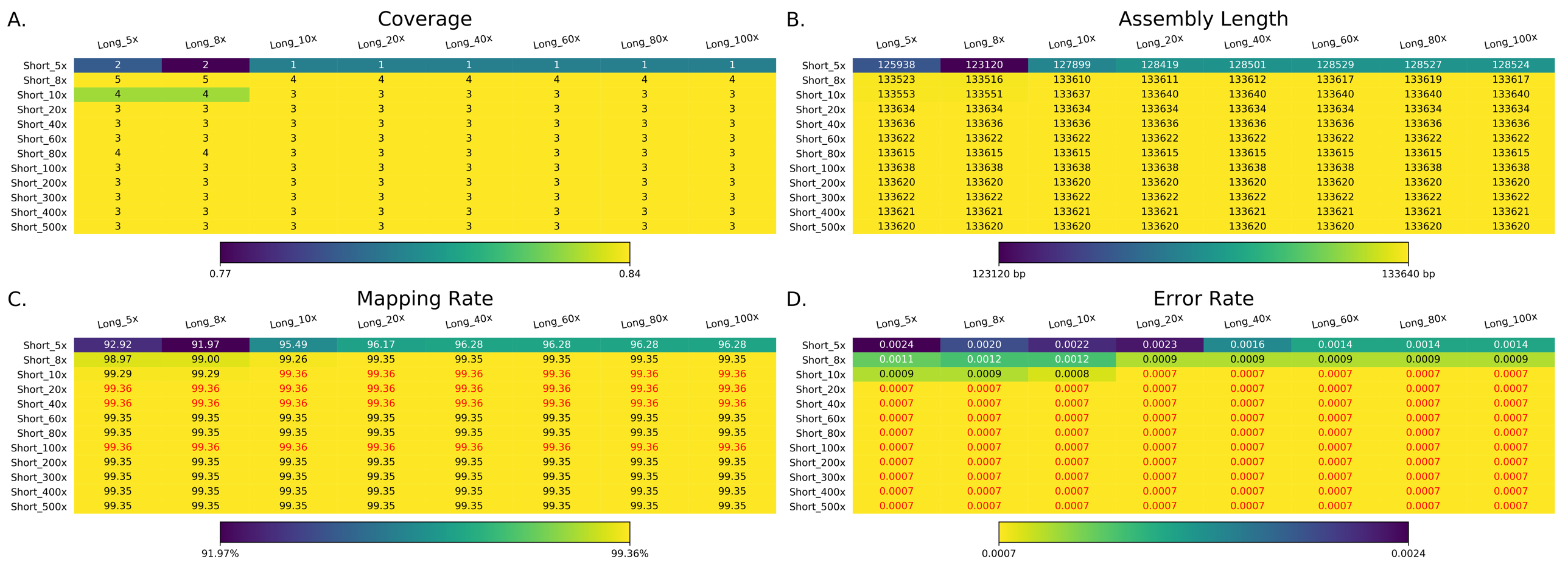

Supplement: Supplementary file 7 — Figure S6. The summary of assemblies with Karect-corrected short-reads and ≤ 10 kb long-reads. Long and Short indicate the coverage of this assembly. A. The total coverage of the chloroplast genome across all contigs output by the assembler. The number is the number of contigs of each assembly, whereas the heatmap is the genome coverage (it could be over 100% if some duplications exist). The heatmap color is reversed compared to the Fig. 1 to make the color in all figure panel A show consistence. B. The assembly length of different assemblies after manual curation (e.g. removing duplicate regions). C. The mapping rate of validation reads to the assemblies after manual curation. Assemblies with highest mapping rate (99.43%) are marked with red. D. The average per-base error rate of validation reads mapped to each manually-curated genome assembly. Assemblies with the lowest error rate (0.0007) are marked with red. (PNG 1210 kb) [file 12864_2018_5348_MOESM7_ESM.png]

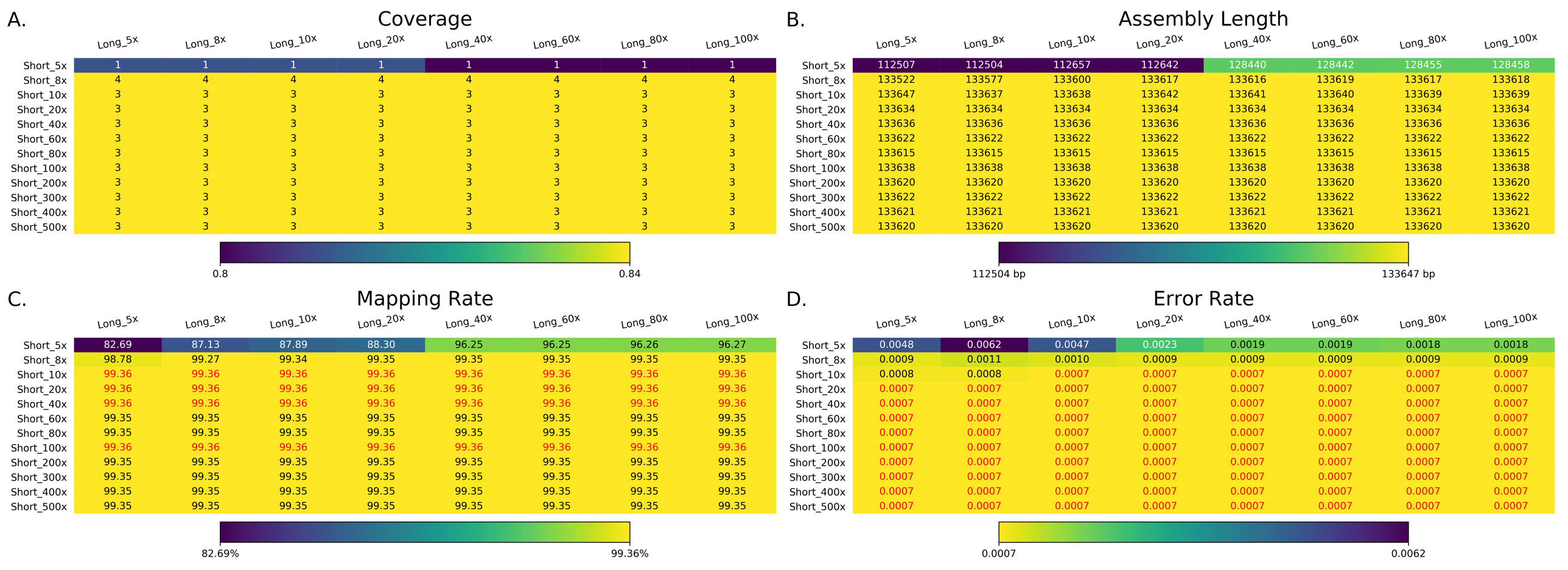

Supplement: Supplementary file 8 — Figure S7. The summary of assemblies with Karect corrected short-reads and 10–20 kb long-reads. Long and Short indicate the coverage of this assembly. A. The total coverage of the chloroplast genome across all contigs output by the assembler. The number is the number of contigs of each assembly, whereas the heatmap is the genome coverage (it could be over 100% if some duplications exist). The heatmap color is reversed compared to the Fig. 1 to make the color in all figure panel A show consistence. B. The assembly length of different assemblies after manual curation (e.g. removing duplicate regions). C. The mapping rate of validation reads to the assemblies after manual curation. Assemblies with highest mapping rate (99.43%) are marked with red. D. The average per-base error rate of validation reads mapped to each manually-curated genome assembly. Assemblies with the lowest error rate (0.0007) are marked with red. (PNG 1190 kb) [file 12864_2018_5348_MOESM8_ESM.png]

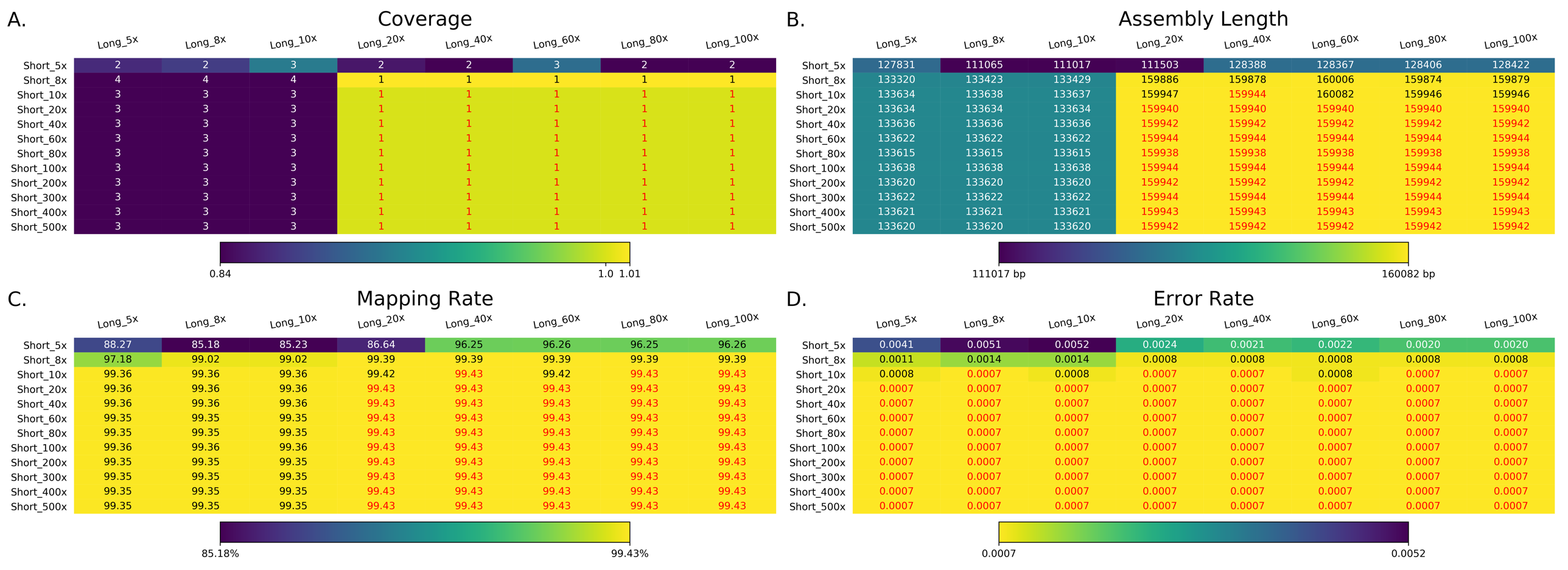

Supplement: Supplementary file 9 — Figure S8. The summary of assemblies with Karect corrected short-reads and 20–30 kb long-reads. Long and Short indicate the coverage of this assembly. A. The total coverage of the chloroplast genome across all contigs output by the assembler. The number is the number of contigs of each assembly, whereas the heatmap is the genome coverage (it could be over 100% if some duplications exist). Numbers marked with red contained a single contig covering the whole chloroplast genome. The heatmap color is reversed compared to the Fig. 1 to make the color in all figure panel A show consistence. B. The assembly length of different assemblies after manual curation (e.g. removing duplicate regions). Numbers marked with red denote assemblies with the expected length, in the range 155,938 bp–155,945 bp. C. The mapping rate of validation reads to the assemblies after manual curation. Assemblies with highest mapping rate (99.43%) are marked with red. D. The average per-base error rate of validation reads mapped to each manually-curated genome assembly. Assemblies with the lowest error rate (0.0007) are marked with red. (PNG 1330 kb) [file 12864_2018_5348_MOESM9_ESM.png]

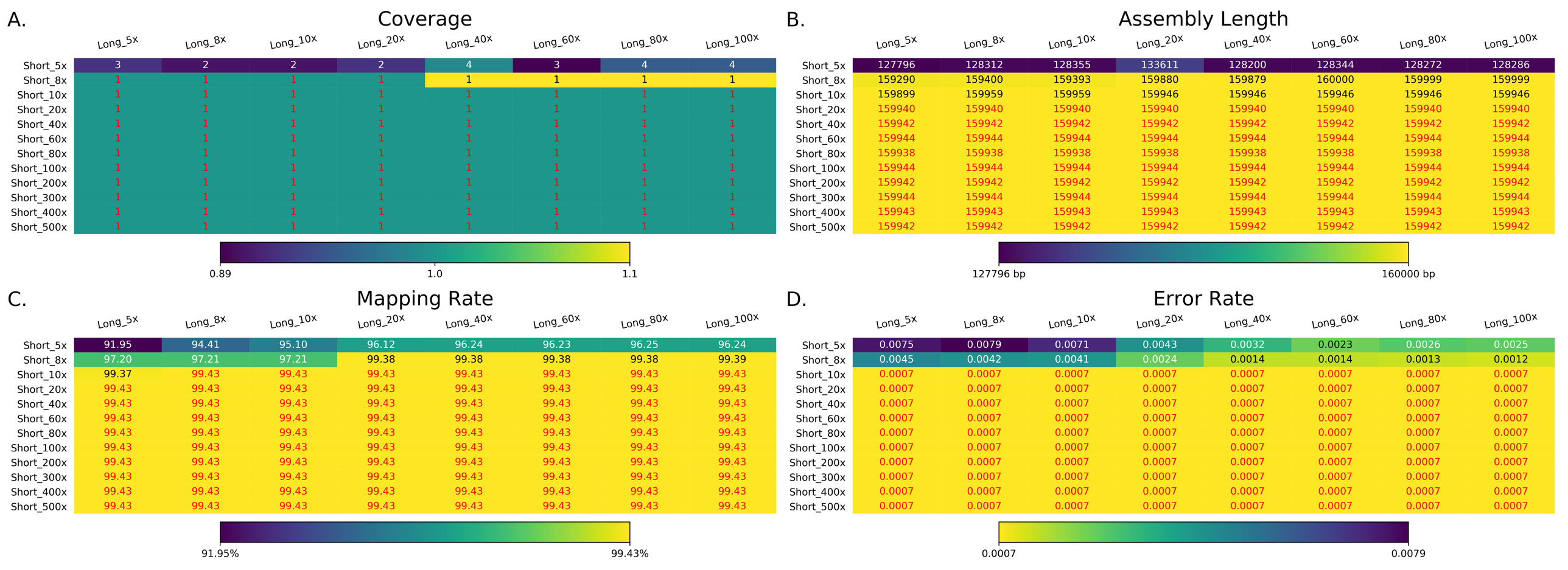

Supplement: Supplementary file 10 — Figure S9. The summary of assemblies with Karect corrected short-reads and 30–40 kb long-reads. Long and Short indicate the coverage of this assembly. A. The total coverage of the chloroplast genome across all contigs output by the assembler. The number is the number of contigs of each assembly, whereas the heatmap is the genome coverage (it could be over 100% if some duplications exist). Numbers marked with red contained a single contig covering the whole chloroplast genome. The heatmap color is reversed compared to the Fig. 1 to make the color in all figure panel A show consistence. B. The assembly length of different assemblies after manual curation (e.g. removing duplicate regions). Numbers marked with red denote assemblies with the expected length, in the range 155,938 bp–155,945 bp. C. The mapping rate of validation reads to the assemblies after manual curation. Assemblies with highest mapping rate (99.43%) are marked with red. D. The average per-base error rate of validation reads mapped to each manually-curated genome assembly. Assemblies with the lowest error rate (0.0007) are marked with red. (PNG 1190 kb) [file 12864_2018_5348_MOESM10_ESM.png]

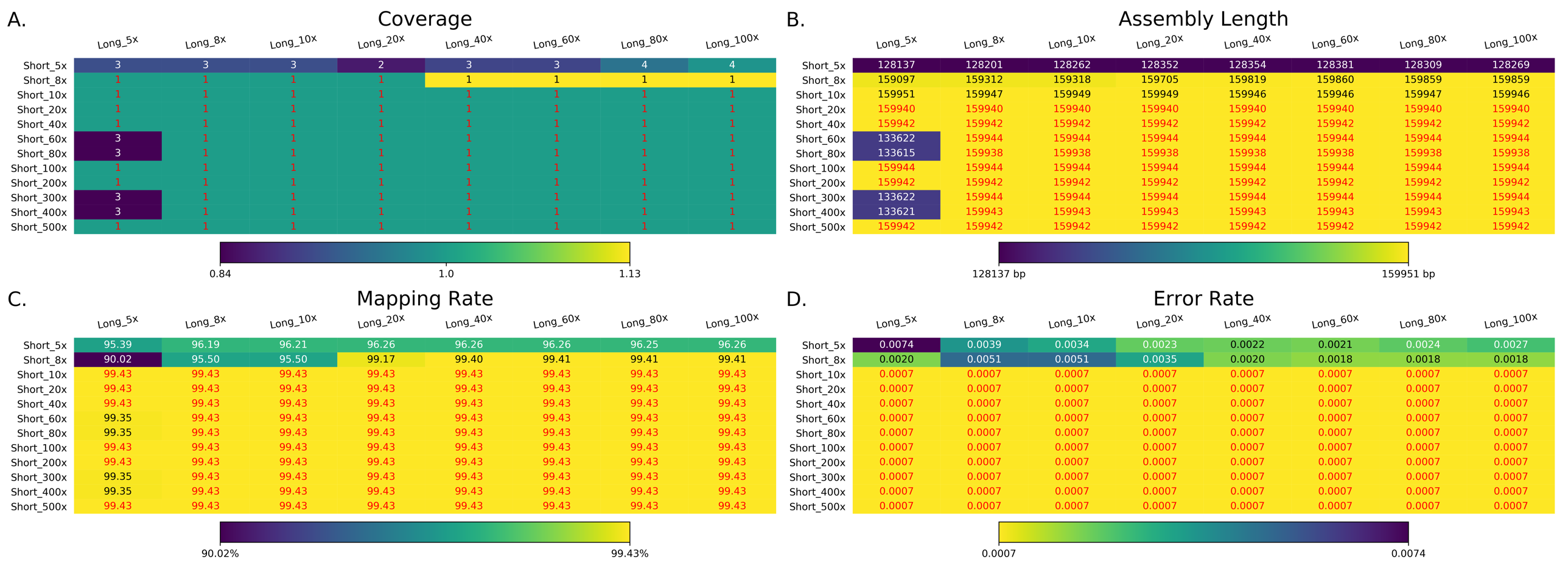

Supplement: Supplementary file 11 — Figure S10. The summary of assemblies with Karect corrected short-reads and 40–50 kb long-reads. Long and Short indicate the coverage of this assembly. A. The total coverage of the chloroplast genome across all contigs output by the assembler. The number is the number of contigs of each assembly, whereas the heatmap is the genome coverage (it could be over 100% if some duplications exist). Numbers marked with red contained a single contig covering the whole chloroplast genome. The heatmap color is reversed compared to the Fig. 1 to make the color in all figure panel A show consistence. B. The assembly length of different assemblies after manual curation (e.g. removing duplicate regions). Numbers marked with red denote assemblies with the expected length, in the range 155,938 bp–155,945 bp. C. The mapping rate of validation reads to the assemblies after manual curation. Assemblies with highest mapping rate (99.43%) are marked with red. D. The average per-base error rate of validation reads mapped to each manually-curated genome assembly. Assemblies with the lowest error rate (0.0007) are marked with red. (PNG 1210 kb) [file 12864_2018_5348_MOESM11_ESM.png]

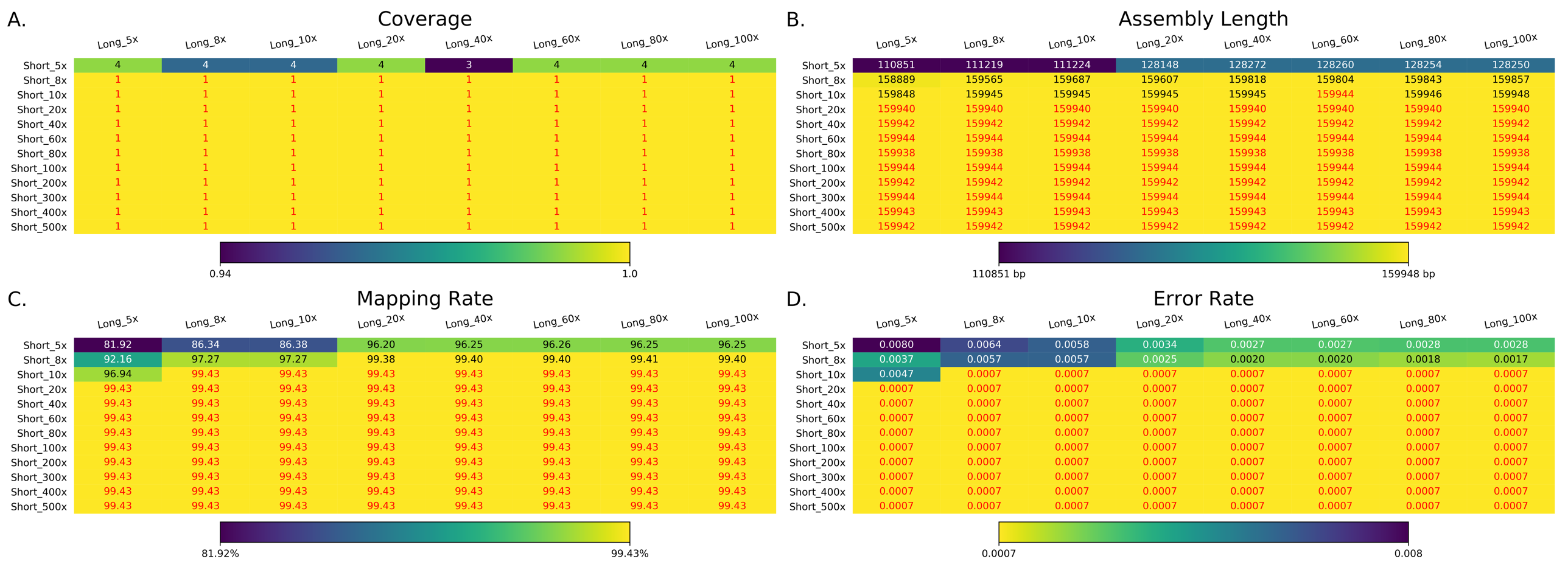

Supplement: Supplementary file 12 — Figure S11. The summary of assemblies with Karect corrected short-reads and ≥ 50 kb long-reads. Long and Short indicate the coverage of this assembly. A. The total coverage of the chloroplast genome across all contigs output by the assembler. The number is the number of contigs of each assembly, whereas the heatmap is the genome coverage (it could be over 100% if some duplications exist). Numbers marked with red contained a single contig covering the whole chloroplast genome. The heatmap color is reversed compared to the Fig. 1 to make the color in all figure panel A show consistence. B. The assembly length of different assemblies after manual curation (e.g. removing duplicate regions). Numbers marked with red denote assemblies with the expected length, in the range 155,938 bp–155,945 bp. C. The mapping rate of validation reads to the assemblies after manual curation. Assemblies with highest mapping rate (99.43%) are marked with red. D. The average per-base error rate of validation reads mapped to each manually-curated genome assembly. Assemblies with the lowest error rate (0.0007) are marked with red. (PNG 1180 kb) [file 12864_2018_5348_MOESM12_ESM.png]

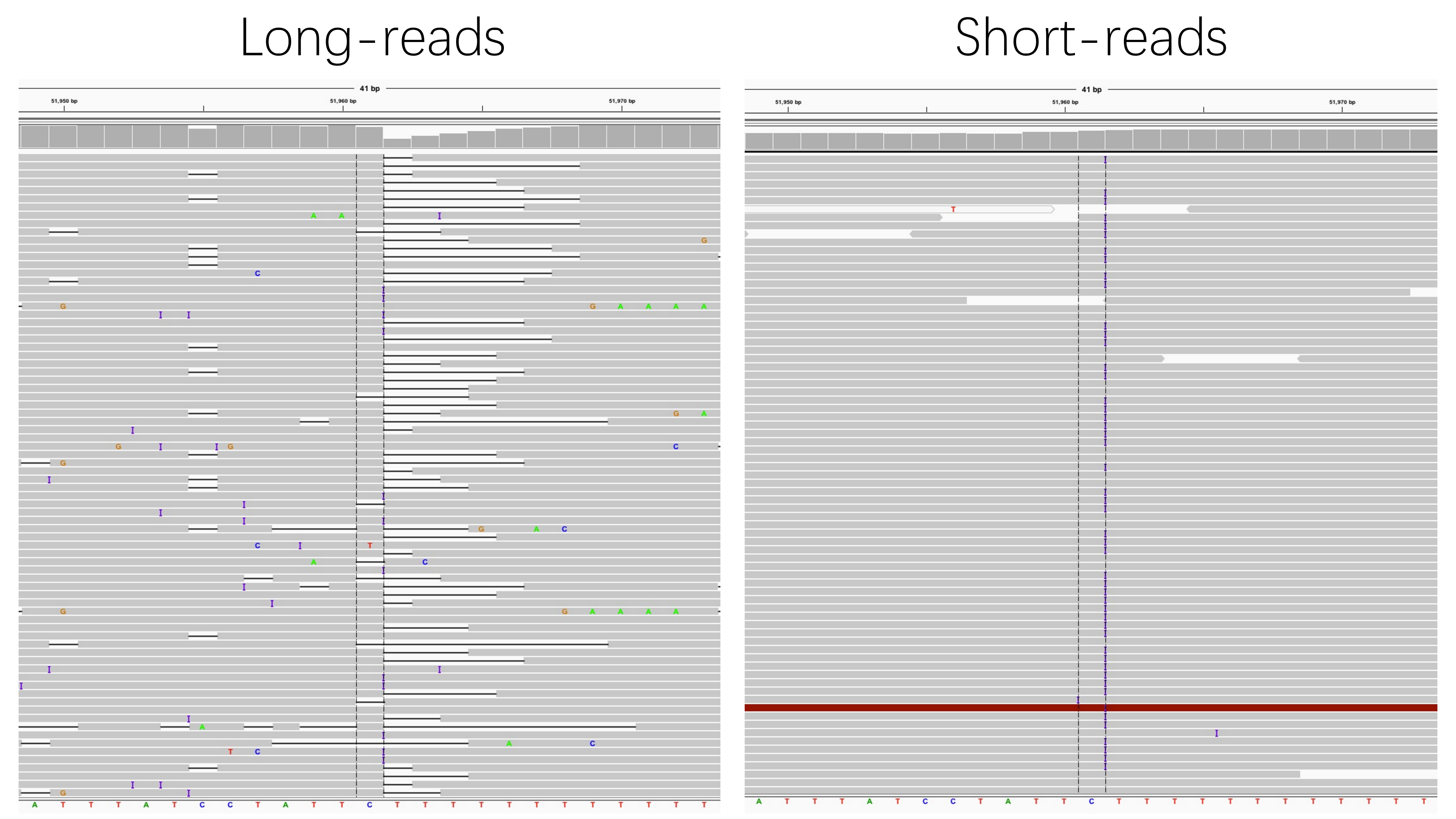

Supplement: Supplementary file 13 — Figure S12. The long−/short-read mapping coverage in the possible heteroplastic site 51,961 (IGV view). The purple “I” in short-reads indicated the T insertion, whereas the black lines in long-reads indicate the deletion during that region. (PNG 978 kb) [file 12864_2018_5348_MOESM13_ESM.png]
