# Supplementary material for: Assembly of chloroplast genomes with long- and short-read data: a comparison of approaches using Eucalyptus pauciflora as a test case
Source: BMC Genomics. 2018 Dec 29;19:977. doi: 10.1186/s12864-018-5348-8 (PMC6311037; doi:10.1186/s12864-018-5348-8)
Supplement: Supplementary file 14 — Supplementary result. (DOCX 13 kb) [file 12864_2018_5348_MOESM14_ESM.docx]

Since Unicycler is not focussed on organelle genome assembly, we tested the performance of one organelle genome specific assembler, NOVOPlasty. We assembled the *E. pauciflora* chloroplast genome with different coverages (Table S2).

At coverages below 200x, the results of NOVOPlasty were highly variable. For example, at 20x coverage (at which coverage Unicycler reliably produced 3 contigs that can be manually combined into a full genome assembly, following the information in the graphical assembly output from Unicycler itself), NOVOPlasty produced a single contig of just 35 kb. At 60x coverage, it produced a full length assembly in a single contig. But at 80x coverage it produced a 2-contig assembly of just 36 kb, and at 100x coverage it produced a 5-contig assembly of 160 kb. At 200x coverage and above, the assemblies varied in length by ~40 bp.

The results from NOVOPlasty were also limited by the input length parameter. When this length was set to a maximum of 140 kb, the maximum length of the assembly was 140 kb. For example, even with 500x coverage, if the maximum length parameter is set to 140 kb, then the resulting assembly is 140 kb. This suggests that NOVOPlasty may be of somewhat limited use in cases where the likely size of the genome is not known, for example in cases where genomes have a non-cannonical structure. This limitation is not shared by Unicycler, which does not make prior assumptions about the length of an assembly.

For these reasons, we do not discuss the results from NOVOPlasty further, but instead use the results from Unicycler to represent the short-read-only assemblies, as these results are more stable and will generalize to a larger range of *de novo* chloroplast genome assembly projects.
